# H-NS controls metabolism and stress tolerance in *Escherichia coli *O157:H7 that influence mouse passage

**DOI:** 10.1186/1471-2180-6-72

**Published:** 2006-08-15

**Authors:** Irfan Erol, Kwang-Cheol Jeong, David J Baumler, Boris Vykhodets, Sang Ho Choi, Charles W Kaspar

**Affiliations:** 1 Department of Food Hygiene and Technology, University of Ankara, Ankara, Turkey; 2 Department of Food Microbiology and Toxicology, University of Wisconsin, Madison, Wisconsin 53706-1187, USA; 3 Cellular and Molecular Biology, University of Wisconsin, Madison, Wisconsin 53706-1187, USA; 4 Department of Food Science and Technology, Seoul National University, Seoul 151-742, South Korea

## Abstract

**Background:**

H-NS is a DNA-binding protein with central roles in gene regulation and nucleoid structuring in *Escherichia coli*. There are over 60 genes that are influenced by H-NS many of which are involved in metabolism. To determine the significance of H-NS-regulated genes in metabolism and stress tolerance, an *hns *mutant of *E. coli *O157:H7 was generated (*hns::nptI*, FRIK47001P) and its growth, metabolism, and gastrointestinal passage compared to the parent strain (43895) and strain FRIK47001P harboring pSC0061 which contains a functional *hns *and 90-bp upstream of the open-reading frame.

**Results:**

The *hns *mutant grew slower and was non-motile in comparison to the parent strain. Carbon and nitrogen metabolism was significantly altered in the *hns *mutant, which was incapable of utilizing 42 carbon, and 19 nitrogen sources that the parent strain metabolized. Among the non-metabolized substrates were several amino acids, organic acids, and key metabolic intermediates (i.e., pyruvate) that limit carbon acquisition and energy generation. Growth studies determined that the parent strain grew in LB containing 14 to 15% bile or bile salts, while the *hns *mutant grew in 6.5 and 9% of these compounds, respectively. Conversely, log-phase cells of the *hns *mutant were significantly (p < 0.05) more acid tolerant than the parent strain and *hns *mutant complemented with pSC0061. In mouse passage studies, the parent strain was recovered at a higher frequency (p < 0.01) than the *hns *mutant regardless of whether log- or stationary-phase phase cells were orally administered.

**Conclusion:**

These results demonstrate that H-NS is a powerful regulator of carbon and nitrogen metabolism as well as tolerance to bile salts. It is likely that the metabolic impairments and/or the reduced bile tolerance of the *E. coli *O157:H7 *hns *mutant decreased its ability to survive passage through mice. Collectively, these results expand the influence of H-NS on carbon and nitrogen metabolism and highlight its role in the ability of O157:H7 strains to respond to changing nutrients and conditions encountered in the environment and its hosts.

## Background

*Escherichia coli *O157:H7 is a human pathogen that causes hemorrhagic colitis in humans and in some cases may incite hemolytic uremic syndrome [1]. Cattle are a principal reservoir and contaminated ground beef is most frequently implicated in foodborne outbreaks associated with this human pathogen [2,3]. Adult cattle harboring serotype O157:H7 strains are asymptomatic, and the numbers and duration of shedding are influenced by a number of factors including feed, the microbial composition of the intestinal tract, the time of year, and environmental sources [4]. The complex interplay between the bovine host and *E. coli *O157:H7 persistence has not been fully elucidated although Naylor et al. [5] recently demonstrated localization and adherence to mucosal epithelium in the recto-anal junction. The changing conditions encountered by *E. coli *O157:H7 in the bovine intestinal tract and the environment require that it closely control metabolism and stress protection.

H-NS is a cytoplasmic protein that is abundant in the *E. coli *nucleoid [6]. It plays a role in the condensation of chromosomal DNA by binding to curved DNA and consequently can influence the expression of many genes either directly or indirectly [7,8]. The level of H-NS in cells remains relatively constant throughout log- and stationary-phases although *hns *transcription is subject to auto repression [9,10]. The production of over 60 proteins in *E. coli *are either transcriptionally or translationally controlled by H-NS [8,9,11]. Several genes modulated by H-NS are responsive to changes in environmental conditions like pH, osmolarity, and temperature [8,9,11-15]. The involvement of H-NS in modulating genes responsive to environmental stimuli make it an important regulator of virulence in several pathogens including *Shigella *and *Salmonella *[12,13,16-19].

Although there has been considerable work on gene regulation by H-NS, there has been comparatively little work on its impact on bacterial metabolism and stress tolerance. Considering the varied and changing environments encountered by *E. coli *O157:H7, a detailed understanding of the consequences of modulators that are responsive to environmental conditions is particularly important. To this end, an *hns *mutant was constructed to define the growth and metabolic changes and any subsequent impact on gastrointestinal passage in this important human pathogen.

## Results

### Generation and confirmation of the *hns::nptI *mutant

A 695-bp fragment that includes the *hns *structural gene and upstream regulatory region was amplified by PCR from genomic DNA of *E. coli *O157:H7 ATCC 43895. The nucleotide sequence of the *Eco*RI-*Bam*HI fragment was determined and was identical to the sequenced gene in *E. coli *O157:H7 EDL933 [20]. Transconjugants resulting from the conjugation of pSC0063 from *E. coli *SM10 λ*pir *to *E. coli *O157:H7 strain ATCC 43895 were kanamycin resistant, ampicillin sensitive, sucrose positive, and O157 positive. Confirmation of a double crossover in which wild-type *hns *was replaced with *hns::nptI *was confirmed by PCR (data not shown). PCR analysis of genomic DNA from the parental strain (ATCC 43895) with primers choi9917 and choi9918 generated a 375-bp fragment; whereas, genomic DNA from the *hns::nptI *mutant (FRIK47001P) resulted in a DNA fragment approximately 1.6-kb in length. The 1.6-kb fragment is in agreement with the projected size of the DNA fragment containing wild-type *hns *(375 bp) and the *nptI *gene (1.2 kb).

### Growth and serological characteristics

The growth of the *hns *mutant (FRIK47001P) in LB at 37°C was slower and reached a lower maximum optical density than the parent strain or FRIK47001P containing pSC0061 (data not shown). The doubling times of the parent, complemented, and mutant strains were 27, 34, and 36 minutes, respectively. The maximum OD_600 _nm achieved by the parent and complemented strains was 1.3 to 1.4 after 18 h of incubation while the *hns *mutant reached a maximum OD_600 nm _= 1.0. Similar to the results in LB, the growth of the *hns *mutant in LB supplemented with mucin or in fecal slurries was slower and reached a lower final density than the parent strain. In addition, the colony size of the *hns *mutant on agar media was smaller than the parent and complemented strains. The presence of kanamycin in the growth media did not influence the growth of the mutant. These results are in agreement with results with *hns *mutants of non-pathogenic *E. coli *which had greater doubling times than the parent strains [11,21]. One contributor to the longer doubling time is the increased σ^s ^level in the *hns *mutant, which is partially suppressed in *hns rpoS *double mutants [11]. The *hns *mutant tested negative for the H7 antigen and was non-motile in motility medium (data not shown); however, the O157 antigen was still detected. The absence of H7 is consistent with previous data showing that H-NS is involved in the control of flagella gene expression and that *hns *mutants are non-motile [22,23].

### Growth inhibition by pH, NaCl, and bile

**Figure 1:**
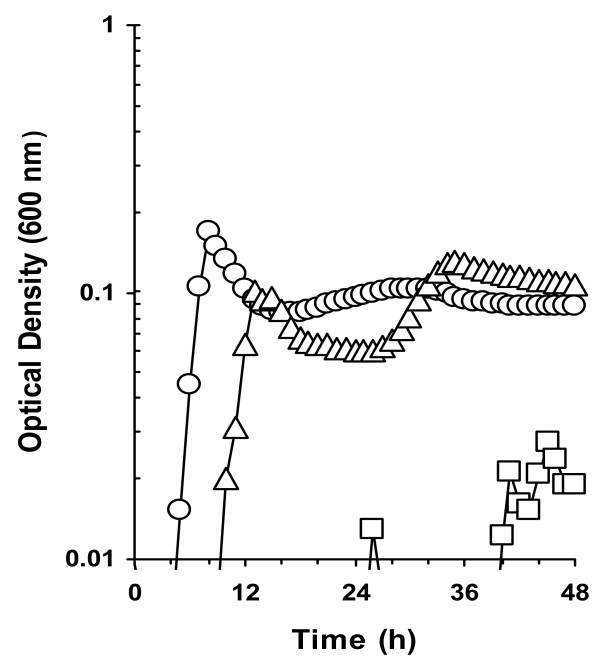
**Effect of bile salts on the growth of *E. coli *O157:H7 43895 (parent) and *hns *mutant (FRIK47001P)**. Growth of the parent strain (○), *hns *mutant (□), and *hns *mutant complemented containing pSC0061 (Δ) in LB containing 5% bile salts number three at 37°C. Data points are the average values from three trials.

In addition to slower growth, the *hns *mutant was more susceptible to inhibition by low pH, NaCl, bile (Table 1) and cefixime (data not shown) compared to the parent and complemented strains. Low temperature further slows the growth of *hns *mutants [24]. Thus, the growth of the mutant was further restricted when low temperature was combined with another inhibitor. The minimum growth pH of the *hns *mutant at the different temperatures was 4.5 to 5.0 which was 0.5 to 1.0 pH unit higher than the parent and complemented strains with the greatest difference observed at the lowest incubation temperature (16.5°C). Both the parent and complemented strains grew at pH 4.0 at each incubation temperature. Similar to the results with pH, the *hns *mutant grew in LB supplemented with a maximum of 4–6% NaCl compared to 6–7% for the parent and complemented strains. The test strains tolerated the highest concentration of NaCl when incubated at 25°C. There was one exception in which the *hns *mutant complemented with pSC0061 grew in a maximum of 4% NaCl at 16.5°C which was equivalent to the maximum level tolerated by the *hns *mutant. It is unclear why the complemented strain differed from the parent strain in this case where in all other growth conditions the results were the same. An unexpected finding was the ability of the *E. coli *O157:H7 to grow in LB containing bile compounds at 14–15%. The *hns *mutant was significantly more sensitive to bile salts and bovine bile preparations and grew in the presence of a maximum of 6.5% and 9.0% of these compounds, respectively. The growth of the strains in the presence of bile was further studied in LB containing 5% bile salts. Although the *hns *mutant grew, it had a greatly extended lag period (approximately 40 h) in comparison to the parent and complemented strains (<12 h) (Figure 1).

### Acid tolerance

**Figure 2:**
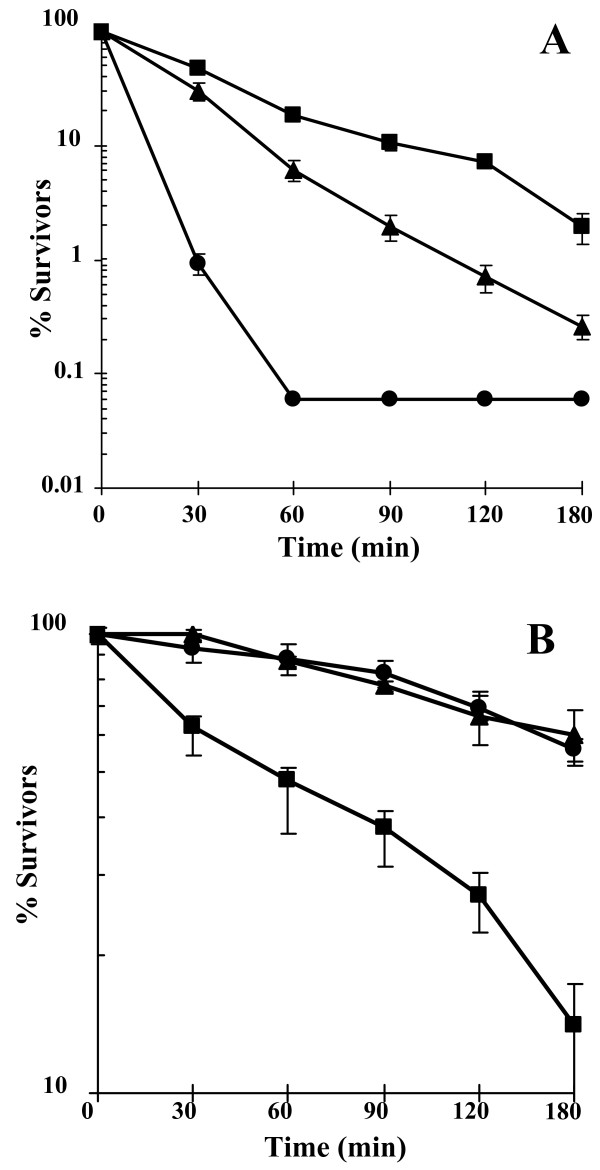
**Acid tolerance of *E. coli *O157:H7 43895 (parent) and *hns *mutant (FRIK47001P)**. Survival of the parent strain (●), *hns *mutant (■), and *hns *mutant complemented containing pSC0061 (▲) in simulated gastric fluid (pH 1.5) at 37°C when using log-phase (A) and stationary-phase (B) cells. Data points are the average values from at least three trials. Error bars represent the standard error of the mean.

The acid tolerance of the parent, *hns *mutant, and complemented strains was tested using both log- and stationary-phase cells. The survival of log-phase cells of the *hns *mutant was significantly greater (p < 0.05) than the parent strain when challenged in synthetic gastric fluid (pH 2.0) (Figure 2A). These findings are consistent with the derepression of σ^s ^(*rpoS*)-regulated genes in the absence of H-NS. Stationary phase increased the acid tolerance compared to log phase in all three strains and the numbers decreased by less than 1 log_10 _CFU/ml after 3 h of acid challenge (Figure 2B). However, the parent and *hns*-complemented strains exhibited similar acid tolerance that was significantly greater (p < 0.05) than the *hns *mutant.

In contrast to the findings with acid challenges conducted in synthetic gastric fluid that is acidified with hydrochloric acid, there was no difference in the survival of the parent and *hns *mutant strains challenged in LB acidified with acetic or lactic acids (data not shown).

### Glutamate decarboxylase activity

In an attempt to discern the reduced acid tolerance in stationary-phase cells of the *hns *mutant, glutamate decarboxylase (GAD) activity was measured (ΔpH/h/10^8 ^CFU). The *hns *mutant had significantly less (p < 0.01) GAD activity than the parent strain and strain FRIK47001P complemented with pSC0061 (p < 0.001) when examining stationary-phase cells (data not shown). These findings provide one possible explanation for the reduced acid tolerance in comparison to the parent strain of the *hns *mutant in stationary phase.

### Sole carbon and nitrogen sources

Metabolic characterization using Phenotype Microarrays showed that the *hns *mutant was incapable of utilizing 42 carbon and 19 nitrogen sources that the parent strain metabolized (Tables 2 and 3). The ability of the *hns *mutant to uptake and/or use a variety of substrates as sole carbon sources was significantly altered. The parent strain was able to use six amino acids as sole carbon sources that the *hns *mutant was unable to utilize. The mutant did not use several disaccharides (rhamnose, lactulose, sucrose, maltose) and trehalose, dulcitol, D-ribose, and dextrin. In addition, eight organic acids, including pyruvate, were not used as a sole carbon sources by the *hns *mutant (Table 3). H-NS is involved in the regulation of genes associated with carbohydrate metabolism, like the maltose operon [25], but there were no carbon sources that were metabolized by the mutant that were not utilized by the parent strain. The parent strain metabolized six amino acids as sole nitrogen sources that the *hns *mutant was unable to use; however, the mutant was capable of using several dipeptides (Table 3). Nitrite was one nitrogen source that was utilized by the *hns *mutant but not the parent strain (data not shown). The findings from this metabolic characterization greatly expand the role of H-NS as a regulator of carbon and nitrogen metabolism in *E. coli*.

### Gastrointestinal passage

Testing of fecal samples prior to inoculation determined that mice were *E. coli *O157:H7 negative and that few bacteria grew on the selective agar that were similar to the inoculation strain (i.e., sorbitol negative). The administration of the parent strain, *hns *mutant (FRIK47001P), and *hns *mutant complemented with pSC0061 did not result in signs of illness in ICR mice. Although mice were tested for 3 days post inoculation, all mice that shed the inoculated *E. coli *O157:H7 strain tested positive one day after inoculation and some mice continued to shed the strain at day 2. No fecal samples tested positive at day 3. The results from three trials are shown in Table 4. The parent strain was recovered from a total of 13 mice of 27 inoculated with log-phase cells (3 trials and 3 inoculation doses) while only a single mouse receiving the *hns *mutant tested positive at a dose of 10^3 ^CFU. The results between the parent and *hns *mutant were statistically different (p < 0.01) at a dose of 10^4^. Similar results were obtained with stationary-phase cells of the parent strain, which was detected in a total of 17 of 27 mice inoculated. The passage of the *hns *mutant was not improved when stationary-phase cells were administered, and the feces from only 1 mouse (dose of 10^4 ^CFU) of 27 inoculated tested positive. At doses of 10^3 ^and 10^4 ^CFU, the results with stationary-phase cells of the parent strain were statistically different (p < 0.05 and p < 0.01, respectively) from those obtained with the *hns *mutant. The passage of the *hns *mutant complemented with a functional *hns *in pSC0061 was similar to that of the parent strain (data not shown). The diminished passage of the *hns *mutant was further demonstrated when the percent of the dose surviving passage through the mouse was calculated. Approximately, 7.9% of the parent strain and 4.2% of the *hns *mutant complemented with pSC0061 were recovered in feces compared to only 0.2% of the *hns *mutant that was determined from the few fecal samples that harbored the mutant. These results demonstrate that H-NS significantly impacts gastrointestinal passage in mice.

## Discussion

*E. coli *O157:H7 must respond to changing environmental conditions whether it is present in the gastrointestinal tract or the environment [26]. Metabolic pathways must be carefully controlled and stress-protection systems triggered in a timely manner to effectively compete and survive. H-NS is a major component of the bacterial nucleoid, having pleiotropic effects on gene expression, genome stability and DNA recombination [9,27]. H-NS is involved in the control of several genes in *E. coli *associated with the metabolism of specific substrates as well as stress protection [8,9,15]; such as Lrp (leucine-responsive regulatory protein), σ^s ^(*rpoS*), and two-component systems (PhoP, EvgA and YedW) [8]. Thus, H-NS is a master regulator affecting the expression of individual genes and regulons with several of the phenotypes observed in *hns *mutants occurring as a result of indirect effects on gene expression [8,27].

**Figure 3:**
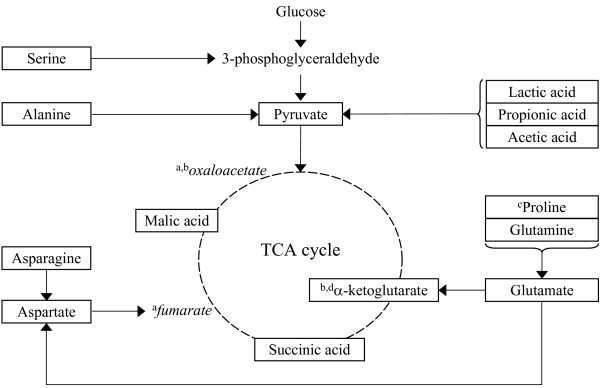
**Key carbon sources that were not metabolized by the *E. coli *O157:H7 *hns *mutant**. Some of the metabolic intermediates and substrates that were not (□, open box) metabolized as sole carbon sources by the *hns *mutant of *E. coli *O157:H7 when examined using Phenotypic Microarrays. Both the parent and *hns *mutant strains metabolized the compounds that are not in boxes. Compounds in italics which were not tested (a). *E. coli *converts glutamate and oxaloacetate to α-ketoglutarate and aspartate (b) [55,56]. Glycyl-L-proline was tested (c). α-ketoglutarate was not metabolized as a sole carbon source by neither the parent strain nor the *hns *mutant (d).

In our studies of an *hns *mutant of *E. coli *O157:H7, the growth was slower than the parent strain. Although it is not completely understood, the increased doubling time in *hns *mutants is partially related to the increased level of σ^s ^and the possibility of secondary site mutations that affect growth [11]. H-NS negatively influences the level of σ^s ^at the posttranscriptional level [9,28,29], and the level of σ^s ^increases in log-phase cells of *hns *mutants [11,30]. Another factor that contributes to the level of σ^s ^in *hns *mutants is the increase in the heat-shock chaperone DnaK which enhances σ^s ^stability [15]. The increase in σ^s ^results in the production of σ^s^-regulated proteins some of which are involved in stress protection [8,15,30]. Results from this study using phenotypic microarrays showed that H-NS has a significant impact on carbon and nitrogen metabolism that likely impact the growth of the mutant (Tables 3 and 4). Among the substrates that were not metabolized were several carbon and nitrogen sources, including several key metabolic intermediates (Figure 3) that likely influence growth and energy generation. It is probable that the slower growth of the *hns *mutant is a result of the combined effects of the increased level of σ^s ^[11] and altered substrate metabolism. However, another possibility that cannot be excluded is the generation of a second site mutation that influenced growth [11,40].

The growth of the *hns *mutant was also more susceptible to inhibition by NaCl and bile salts than the parent strain (Table 1). These differences were accentuated by sub-optimal growth temperature, often encountered when outside of the intestinal tract. A significant finding from these growth comparisons was the high tolerance of the parent *E. coli *O157:H7 strain to bile and bile salts (15 and 14%, respectively), while the growth of the *hns *mutant was limited to a maximum of 9.0 and 6.5%, respectively. Bile salts are produced by the liver to breakdown fat and are deleterious to biological membranes. *E. coli *must tolerate bile salts that can reach concentrations of 20 mM in the duodenum and even higher in the small intestine [31]. The reduced capacity of the mutant to tolerate bile salts might be a consequence of membrane changes in the *hns *mutant. H-NS is known to influence expression of at least 5% of the genes in *E. coli*, many that encode for membrane proteins [8]. The possibility of an altered membrane is supported by the inhibition of the mutant by the cephalosporin-like antibiotic, cefixime, while the parent strain grew in media containing 0.4 μg cefixime/ml. Future studies with H-NS-deficient strains should be useful in identifying the proteins involved in bile tolerance in *E. coli *O157:H7 strains.

Acid tolerance is one characteristic that influences the persistence of *E. coli *O157:H7 shedding in cattle [32] and may promote survival during gastric passage in humans [33]. The increased acid tolerance of the *hns *mutant in comparison to the parent strain in log-phase cells is consistent with the expression of σ^s^-regulated proteins [9]. In stationary-phase cells, the acid tolerance increased in all three strains (parent, *hns *mutant, and *hns *complemented strains) in comparison to log-phase cells. However, the *hns *mutant was less acid tolerant than the parent strain in stationary phase. The *gad *system comprised of GadA and GadB, two isoforms of glutamate decarboxylase, and *gadC *that encodes for a glutamate/γ-aminobutyrate antiporter [34-38] is one contributor to acid tolerance. Results from the phenotype microarrays showed that the *hns *mutant did not utilize glutamate as a sole carbon source; therefore, we compared glutamate decarboxylase activity in stationary-phase cells of the mutant and parent strains. The specific activity of glutamate decarboxylase was reduced in the *hns *mutant (p < 0.01) compared to the parent strain. The decreased specific acitivity of glutamate decarboxylase may explain the difference in acid tolerance between the *hns *mutant and parent strain in stationary-phase cells.

In mouse passage studies, recovery of the *hns *mutant was significantly less than the parent strain (Table 2). The mutant was recovered from a single mouse in each of three trials conducted with log-phase and stationary-phase cells. It is possible that the reduced ability of the *hns *mutant to survive mouse passage is a consequence of a depleted energy supply, resulting from its limited capacity to metabolize available substrates, which is necessary for the optimal functioning of homeostasis or stress protection systems (i.e., ATPase). Gene expression profiles of mucus-grown *E. coli *have identified genes involved in the catabolism of gluconate, N-acetylglucosamine, sialic acid, glucuronate, mannose, fucose, and ribose are induced and considered preferred carbon sources [39]. Mutants incapable of metabolizing these seven carbohydrates have a decreased capacity to colonize streptomycin-treated mice. Although the mouse passage assay employed in this study did not evaluate colonization, the results from Phenotype Microarrays found that five of the seven carbohydrates identified by Chang et al. [39] as preferred carbon sources were utilized by the *hns *mutant while the *E. coli *O157:H7 parent strain used all seven carbon sources (see Tables 2 and 3). In addition, the *hns *mutant grew in mucin-supplemented LB broth and fecal slurries to a concentration essentially equivalent to that of the parent strain although the lag-phase was longer and the growth rate slower than that of the parent strain which is similar to the results obtained in growth studies conducted in LB. Therefore, a defect in the ability to utilize a mucin carbohydrate is probably not the reason for the reduced passage of the *hns *mutant.

There are a number of possible antimicrobial factors in the gastrointestinal tract that may be responsible for the diminished capacity of the *hns *mutant to survive passage through ICR mice. Acetate can reach a concentration of 70 mM in the intestinal tract [41,42]. Although log-phase cells of the mutant were more acid tolerant in challenges conducted with hydrochloric acid, additional challenges were conducted with acetic and lactic acids because results from phenotype microarrays found that the mutant did not utilize acetate or lactate as sole carbon sources. It was reasoned that tolerance to these acids might be impaired, but acid challenges with lactic acid and acetic acid at pH 4.4 found no difference in the survival of the parent and *hns *mutant strains with log- or stationary-phase cells (data not shown). Another possibility is that bile salts may have affected passage of the *hns *mutant through mice. In addition to being inhibitory, bile in association with H-NS is known to repress virulence genes [43]. However, additional experiments are needed with bile-sensitive mutants and measurements of bile acid concentrations in the mouse intestinal tract to confirm that bile was responsible for the reduced recoveries of the *hns *mutant. Considering that the intestinal tract contains at least 500 bacterial species [39], it is also possible that one of the commensal microbes produces an antimicrobial factor that is detrimental to the *hns *mutant. Studies with gnotoxenic mice would be particularly valuable in addressing the contributions of competing flora on the survival of the *hns *mutant. Based upon the results from this study, the most plausible explanation for the reduced passage of the *hns *mutant through mice is the regulatory impairments that influence growth and stress protection.

## Conclusion

Results from this study significantly expand the importance of H-NS in carbon and nitrogen metabolism (Figure 3) as well as stress tolerance that collectively influence the ability of *E. coli *O157:H7 to respond to changing conditions it encounters in its host and the environment. This is the first report of the high tolerance of *E. coli *O157:H7 strains to bile and bile salts that may be of significance in localization and persistence within its bovine host and possibly during human infection. Studies are in progress to identify, which proteins that are influenced by H-NS contribute to bile tolerance. The results from this study also establish H-NS as a potential target for control of this important human pathogen.

## Methods

### Bacterial strains and culture conditions

The bacterial strains and plasmids used in this study are listed in Table 5. *E. coli *were grown in Luria-Bertani (LB) broth at 37°C. Antibiotics (Sigma Chemical Company, St. Louis, MO) were added to media when appropriate: ampicillin, 100 μg/ml; kanamycin, 50 μg/ml; and tetracycline, 10 μg/ml. For log-phase and stationary-phase cultures, 5 μl of an overnight culture was used to inoculate 5 ml of LB and incubated with shaking (150 rpm) at 37°C to an optical density at 600 nm = 0.5 (log phase, ~10^8 ^CFU/ml) and optical density at 600 nm = 1.1 (stationary phase, ~10^9 ^CFU/ml), respectively.

### Generation and confirmation of an *hns::nptI *mutant

A DNA fragment containing the *hns *structural gene and flanking regions was amplified from genomic DNA of *E. coli *O157:H7 ATCC 43895 by PCR using oligonucleotide primers (choi0005, 5'-TTTGGATCCAAAGCCTGGCTTGAAGAAGAGATG-3' and choi0006, 5'-TTTGAATTCTTTTGAATTCCTTACATTCCTGGC-3') containing an *Eco*RI (choi0005) and *Bam*HI (choi0006) site on the respective 5'-ends. After PCR amplification, the PCR fragment was digested with *Eco*RI and *Bam*H1 and ligated into pBR322, previously digested with the same enzymes, to produce pSC0061. The nucleotide sequence of the cloned fragment was determined (University of Wisconsin-Biotechnology Center). The suicide vector, pCVD442, was used to generate *hns *mutant in *E. coli *O157:H7 ATCC 43895 by homologous recombination. The *hns *gene in pSC0061 was inactivated by insertion of *nptI *encoding for aminoglycoside 3'-phosphotransferase (confers resistance to kanamycin) digested from pUC4K [44] using *Pst*I and gap filled with Klenow fragment. *npt*I was ligated with a partial *Pst*I digest of pSC0061 carrying *hns *which has a *Pst*I site present within its open reading frame (ORF). The construct (pSC0062) was digested with *Eco*R1 and *Bam*H1 to liberate the 1.9-kb *hns::nptI *fragment. The fragment was blunt-ended with Klenow and ligated with *Sma*I-digested pCVD442 [45] to produce pSC0063. The constructed vector was conjugally mobilized from *E. coli *SM10 λ *pir *[46] carrying the *tra *gene to recipient strain *E. coli *O157:H7 43895. Conjugation and mutant selection were conducted as described previously [47]. Transconjugants that were kanamycin resistant, ampicillin sensitive and tested positive for the O157 antigen by latex agglutination (Oxoid, Basingstoke, England) were selected for further study. The primers choi9917 (5'-ATGAGCGAAGCACTTAAAATTCTGAAC-3') and choi9918 (5'-TTCTTCCATTGCTTTTTTGATTACAGC-3') were used to amplify a portion of the *hns *ORF and confirm the presence of *nptI *in *hns *(1.6-kb fragment) (data not shown). FRIK 47001 (*hns::npt*I) was complemented by transforming FRIK 47001 with pSC0061.

### Phenotype MicroArrays

The analysis of sole carbon and nitrogen source utilization was conducted with Phenotype MicroArrays™ (Biolog, Hayward, Calif.). This method is used to identify the function and global interactions of a gene on cellular metabolism [48,49]. The parent *E. coli *O157:H7 strain ATCC 43895 and *hns::npt*I mutant (FRIK47001) were evaluated for their ability to use carbon and nitrogen sources using PM1, PM2, and a PM3 plates, respectively. Strains were grown on R2A agar (Biolog) overnight at 37°C and sufficient cells were transferred to inoculating fluid to equal the 85%T turbidity standard (Biolog). FRIK47001 was resuspended in inoculating fluid containing glucose rather than succinic acid when inoculating the PM3 plate because it was unable to use succinic acid as a carbon source. Each well was inoculated with 100 μl of the standardized cell suspension, incubated at 37°C for 48 h, and the results recorded.

### Growth studies

Growth studies were conducted in LB adjusted to the appropriate pH or supplemented with NaCl, bile, bile salts, or cefixime using the parent, *hns *mutant, and *hns *mutant complemented with pSC0061. Cells were used to inoculate the adjusted LB medium at a final concentration of 10^5 ^CFU/ml and incubated at 16.5, 25, and/or 37°C without shaking. The minimum pH for growth was determined in the pH range of 3.5 to 7.0 at 0.5 increments. The following were added to LB at the following ranges: NaCl, 0 to 10% (0.5% increments); bile salts no.3 (Difco), 0 to 16%; bovine bile (Sigma), 0 to 16%; porcine stomach mucin (Sigma), 2.5 mg/ml; bovine submaxillary gland mucin (Sigma), 2.5 mg/ml; and cefixime (Lederle), 0.2 to 0.8 μg/ml. Inoculated tubes were incubated at the designated temperature and observed daily for turbidity for up to 21 days. The growth of the strains in the presence of bile salts and mucin were studied in more detail. Growth in LB supplemented with 5% bile salts or 2.5 mg mucin/ml was monitored spectrophotometrically at 600 nm using a Bioscreen Analyzer at 37°C for 48 h (Labsystems, Helsinki, Finland). Plates were shaken for 30s prior to each optical density reading. The growth of strains was also monitored in fecal slurries (mouse feces diluted 1:10 in sterile deionized water). The fecal slurries were inoculated at a final concentration of approximately 10^5 ^CFU/ml from overnight cultures grown in LB, and then incubated at 37°C. Samples were removed and the number of CFU/ml determined by plating on MacConkey sorbitol agar (MSA, Difco) supplemented with 2.5 μg potassium tellurite/ml (Fisher) and 0.05 μg cefixime/ml (Lederle Laboratories, Pearl River, NY) [50] and enumerating the number of sorbitol-negative colonies.

### Acid challenge

Acid tolerance was assessed in synthetic gastric fluid adjusted to pH 1.5 and LB acidified with acetate or lactate (pH 4.4) as previously described [51]. Log (OD_600 _= 0.5) and stationary (OD_600 _= 1.1) phase cells were used to inoculate flasks containing 50 ml of synthetic gastric fluid or acidified LB to achieve a final concentration of ca. 10^5 ^CFU/ml. Flasks were incubated at 37°C with shaking (150 rpm). Samples were removed periodically and then plated on tryptic soy agar (TSA, Difco) using a Model D Spiral Plater (Spiral Systems Inc., Cincinnati, OH). The number of colony forming units (CFU) was determined after 24 h of incubation at 37°C. The survival curves shown are the mean values from at least three trials.

### GAD activity

The cells were examined for GAD activity as previously described [52,53] with the following modifications. Strains were cultured overnight in LB at 37°C for 21 h. The cells from 1.0 ml were pelleted by centrifugation and washed with 0.9% NaCl. The cells were resuspended in 3.0 ml of GAD reagent (1 g of L-glutamic acid, 0.05 g of bromocresol green, 90 g of NaCl, and 3 ml of Triton X-100 per liter) and incubated for 3 h at 35°C. The color and pH change of the reaction mixture was recorded every 30 minutes. GAD activity was reported as the ΔpH/h/10^8 ^cells. The mean values were calculated from at least three trials.

### Mouse inoculation studies

The passage studies were conducted as previously described [32]. In brief, strains were grown in LB at 37°C to log and stationary phase. After harvesting and washing with PBS, the cells were resuspended in 10% sucrose or phosphate buffer (0.01 M, pH 7.4) to a concentration of 10^4^, 10^3^, or 10^2 ^CFU/ml and administrated to individually housed ICR mice (ca. 20 g, Harlan Sprague Dawley Inc., Madison, Wis). Three trials were conducted, and in each trial three mice were inoculated with each concentration of the test strain. Fecal samples were collected each day for 3 days after inoculation and tested for the presence or absence of *E. coli *O157:H7. Fecal samples were diluted (1:10) in modified EC broth supplemented with novobiocin (20 μg/ml) and incubated at 37°C with shaking (150 rpm) for 18–24 h. Following enrichment, 20 μl of Dynabeads^® ^anti-*E. coli *O157 (Dynal Biotech ASA, Oslo, Norway) was added to 0.5 ml of the enriched fecal culture and wash buffer (0.5 ml), incubated, and washed as described by the manufacturer. The resuspended beads were streaked on MacConkey sorbitol agar supplemented with potassium tellurite/ml and cefixime/ml [50]. The plates were incubated at 42°C for 24 h and examined for sorbitol-negative *E. coli *O157:H7 colonies. Suspect colonies were confirmed as O157 by agglutination (*E. coli *O157:H7 test kit, Oxoid). The minimum detection level of this procedure was approximately 100 CFU/g.

### Statistical analyses

The data presented are the mean values from at least three trials. The acid tolerance of strains was compared using the inverse of slopes from linear regression plots of survivor curves. The data from acid challenges were analyzed for statistical differences by one-way analysis of variance. A z-test for was used to evaluate significant differences in the proportions of mice testing positive between inoculation strains [54]. All calculations were conducted using SigmaStat software (Jandel Scientific, San Rafael, Calif.).

## Authors' contributions

IE, KCJ, DJB, and BV conducted laboratory experiments, analyzed data, and contributed to the writing of the manuscript. SHC contributed to the initial concept of this project and constructed and confirmed the *hns *mutant and pSC0061. CWK designed and coordinated the project and wrote the manuscript.

## Figures and Tables

**Table 1:** Growth of *Escherichia coli *O157:H7 strains in LB as influenced by pH, temperature, NaCl, and bile compounds.

	Growth in LB		
	
Growth conditions	ATCC 43895 (parent strain)	FRIK47001P (*hns *mutant)	FRIK47001P (pSC0061)
pH^a^			
37°C	4.0	4.5	4.0
25°C	4.0	4.5	4.0
16.5°C	4.0	5.0	4.0
NaCl (%)^b^			
37°C	6.0	5.0	6.0
25°C	7.0	6.0	7.0
16.5°C	6.0	4.0	4.0
			
Bile salts (%)^c^	14.0	6.5	14.0
Bile (%)^d^	15.0	9.0	15.0

^a^Minimum pH at which growth occurred. LB adjusted to appropriate pH with 5 N HCl.

^b^Maximum percent NaCl in LB at which growth occurred.

^c^Bile salts No. 3 (Difco). Maximum percent in LB that growth occurred at 37°C.

^d^Bovine bile (Sigma). Maximum percent in LB that growth occurred at 37°C.

**Table 2:** Sole carbon source metabolism by the *E*. coli O157:H7 parent (ATCC 43895) and *hns *mutant (FRIK 47001) strains.

Carbohydrates and derivatives	Amino acids and derivatives	Organic acids	Other
**Utilized by the parent strain but not the *hns *mutant**			
Dextrin	D-Alanine	Acetic acid	
Dulcitol	L-Alanine	Formic acid	
3-O-β-D-Galactopyranosyl-D-arabinose	L-Alaninamide	L-Glutamic acid	
L-Galactonic acid-lactone	L-Asparagine	D-Glucuronic acid	
α-methyl-D-Galactoside	glycyl-L-Aspartic acid	L-Lactic acid	
β-methyl-D-Galactoside	glycyl-L-Glutamic acid	D-Malic acid	
β-methyl-Glucoside	L-Glutamine	L-Malic acid	
D-L-αGlycerol phosphate	L-alanyl-Glycine	D, L-Malic acid	
Lactulose	glycyl-L-Proline	Melibionic acid	
Maltose	L-Serine	Mucic acid	
N-acetyl-β-D-Mannosamine		Propionic acid	
L-Rhamnose		Putrescine	
D-Ribose		Pyruvic acid	
Sucrose		methyl Pyruvate	
Succinic acid		bromo Succinic acid	
D-Trehalose		mono methyl Succinate	
**Utilized by the parent and *hns *mutant strains**			
L-Arabinose		D-Galacturonic acid	Adenosine
L-Fucose		D-Gluconic acid	2-deoxy Adenosine
D-Fructose		N-acetyl-Neuraminic acid	N-acetyl-D-Galactosamine
Fructose-6-phosphate			N-acetyl-D-Glucosamine
D-Galactose			Inosine
α-methyl-D-Galactoside			Thymidine
Glucose-1-phosphate			Uridine
Glucose-6-phosphate			
α-D-Glucose			
Glycerol			
α-D-Lactose			
Maltotriose			
D-Mannitol			
D-Mannose			
D-Melibiose			
D-Psicose			
D-Raffinose			
D-Xylose			

**Table 3:** Sole nitrogen source metabolism by the *E*. coli O157:H7 parent (ATCC 43895) and *hns *mutant (FRIK 47001) strains.

Amino acids and derivatives	Peptides	Other
**Utilized by the parent strain but not the *hns *mutant**		
D-Alanine	Ala-His	Alloxan
L-Alanine	Ala-Leu	γ-amino-N-Butyric acid
L-Arginine	Ala-Thr	Cytosine
L-Cysteine		N-acetyl-D-Galactosamine
Glycine		D-Glucosamine
L-Proline		N-acetyl-D-Glucosamine
L-Tryptophan		Glucuronamide
		N-acetyl-D-Mannosamine
		L-Ornithine
		Xanthine
		Xanthosine
**Utilized by the parent and *hns *mutant strains**		
L-Aspartic Acid	Ala-Asp	Adenine
L-Asparagine	Ala-Gln	Adenosine
L-Glutamic Acid	Ala-Glu	Ammonia
L-Glutamine	Ala-Gly	Cytidine
L-Serine	Gly-Asn	
	Gly-Gln	
	Gly-Glu	
	Gly-Met	
	Met-Ala	

**Table 4:** Recovery of *E. coli *O157:H7 strains ATCC43895 (parent) and FRIK47001P (*hns *mutant) from feces following oral administration to ICR mice.^a^

*E. coli *dose (CFU)	No. of mice with a positive fecal sample for *E. coli *O157:H7 in each trial with three mice inoculated per trial (total no. of positive mice/9 mice examined in 3 trials)					
	
	Trial with log-phase cells			Trial with stationary-phase cells		
	
	I	II	III	I	II	III
Strain 43895 (parent)						
10^2^	0	0	1 (1)	0	1	3 (4)
10^3^	2	0	3 (5)	0	2	3 (5)
10^4^	3	1	3 (7)	3	3	2 (8)
						
Strain 47001P (*hns *mutant)						
10^2^	0	0	0 (0)	0	0	0 (0)
10^3^	1	0	0 (1)	0	0	0 (0)^b^
10^4^	0	0	0 (0)^c^	1	0	0 (1)^c^

^a^Cell pellets of the parent and *hns *mutant strains were resuspended and diluted to the appropriate cell number in 10% sucrose. Each mouse was administered 0.5 ml of the sucrose solution containing the designated dose of cells. Results are from fecal pellets collected 12–14 h after oral administration.

^b^Significantly different (p < 0.05) from results with the parent strain at a dose of 10^3 ^CFU.

^c^Significantly different (p < 0.01) from results with the parent strains at a dose of 10^4 ^CFU.

**Table 5:** Plasmids and *E. coli *strains used in this study.

Strain or plasmid	Relevant characteristics^a^	Source or reference
Strains		
DH5α	*supE44 ΔlacU169 *(ϕ80 *lacZ *ΔM15)*hsdR17 recA1 endA1 gyrA96 thi-1 relA1*	Lab collection
ATCC 43895	*stx*_1 _and *stx*_2_; other designation EDL 933W	Lab collection
SY327 λ*pir*	*Δ*(*lac pro*) *argE*(Am) *recA56 nalA *Rif(λpir); π protein for R6K γ *ori*	[57]
SM10 λ*pir*	*thi thr leu tonA lacY supE recA*::RP4-2-Tc::Mu, λ*pir*, *oriT *of RP4; Km^r^	[57]
FRIK47001P^b^	ATCC 43895, *hns::nptI*	This study
Plasmids		
pBR322	Ap^r ^and Tc^r^	Lab collection
pUC4K	pUC4 with *nptI*; Ap^r^, Km^r^	Pharmacia, [44]
pCVD442	R6K γ *ori*, *sacB*, *oriT *of RP4; Ap^r^	J. Kaper, [45]
pSC9916	pBR322 containing Ap^r^, Tc^s^	[47]
pSC0061	pSC9916 containing *hns*; Tc^s^, Ap^r^	This study
pSC0062	pSC0061, *hns::nptI*	This study
pSC0063	pCVD442 containing *hns::nptI*	This study

^a ^Ap^r^, ampicillin resistant; Ap^s^, ampicillin sensitive; Km^r^, kanamycin resistant; Tc^r^, tetracycline resistant; Tc^s^, tetracycline sensitive. Antibiotics were added to media when appropriate at the following concentrations: ampicillin 100 μg/ml, kanamycin 50 μg/ml, tetracycline 10 μg/ml.

^b ^FRIK47001P *hns::nptI *in the parallel orientation.
